# Perianesthetic death: a 10-year retrospective observational study in a Japanese university hospital

**DOI:** 10.1186/s40981-020-0314-2

**Published:** 2020-02-05

**Authors:** Mariko Sato, Mitsuru Ida, Yusuke Naito, Masahiko Kawaguchi

**Affiliations:** Department of Anesthesiology, Nara Medical University, 840 Shijo-cho, Kashihara, Nara, 634-8522 Japan

**Keywords:** Anesthesia, Mortality, Perioperative period

## Abstract

**Background:**

Studies reporting on perianesthetic death and anesthesia-related death are limited. The present study aimed to assess the incidence of perianesthetic death and its relation to anesthesia and to describe the patient characteristics and main events leading to death in cases of anesthesia-related death and anesthesia-contributory death.

**Methods:**

We conducted a retrospective chart review of patients in whom anesthesia procedures were performed by anesthesiologists at a Japanese tertiary hospital between January 2008 and December 2017. Perianesthetic death was defined as death occurring within 48 h of an anesthetic, and it was divided into the following three categories: anesthesia-related death, anesthesia-contributory death, and nonanesthesia-related death. Patient demographics and perioperative factors were analyzed in cases of anesthesia-related death and anesthesia-contributory death.

**Results:**

Among 46,378 patients who underwent anesthetics, 41 experienced perianesthetic death, with an incidence of 8.8/10,000 anesthetics (95% confidence interval [CI], 6.1–11.6). No patient experienced anesthesia-related death, whereas 10 experienced anesthesia-contributory death, with an incidence of 2.1/10,000 (95% CI, 0.69–3.6), and 31 experienced nonanesthesia-related death, with an incidence of 6.8/10,000 (95% CI, 4.2–9.1). The events leading to anesthesia-contributory death were hypovolemia, myocardial infarction, arrhythmia, and respiratory failure, and they occurred during anesthesia maintenance in 5 patients and after surgery in 5 patients.

**Conclusions:**

The incidence of perianesthetic death was 8.8/10,000 anesthetics; however, anesthesia-related death was not detected. Ten patients experienced anesthesia-contributory death, and hypovolemia during or after surgery was most frequently associated with anesthesia-contributory death.

## Introduction

Postoperative death, which is defined as death within 30 days of surgery, accounts for 7.7% of all deaths globally, and surgery is the third most common contributor to death [1]. In particular, death within 48 h of an anesthetic is defined as perianesthetic death, and it is a serious issue among anesthesiologists [2, 3].

Studies focusing on perioperative cardiac arrest have been published; however, studies focusing on perianesthetic death and anesthesia-related death are limited [3–9]. Two analyses of anesthesia-related death based on closed claims analysis and death certificates have been reported, but both analyses failed to provide denominators, making interpretation of the percentage of perianesthetic death difficult [8, 9]. One retrospective analysis of the quality assurance database of a community-based anesthesiology group practice revealed that the perianesthetic death rate and anesthesia-related death rate were 7.53/10,000 cases and 0.05/10,000 cases, respectively [3]. However, these rates vary depending on the institution and the study population, and limited information is available on the incidence of perianesthetic death and its relation to anesthesia in Japan.

Therefore, in the present study, we aimed to assess the incidence of perianesthetic death and its relation to anesthesia at a Japanese tertiary hospital and to describe the patient characteristics and main events leading to death in cases of anesthesia-related death and anesthesia-contributory death.

## Methods

### Ethical approval

This retrospective observational study was approved by the Institutional Review Board of Nara Medical University (Kashihara, Nara, Japan; Chairperson Prof. M. Yoshizumi; approval no. 2964; December 17, 2018). The requirement of informed consent was waived because of the retrospective nature of this study.

### Patient selection

We included patients who underwent anesthetics performed under general and local anesthesia managed by anesthesiologists at Nara Medical University between January 2008 and December 2017. We excluded patients who underwent anesthesia procedures performed in the emergency room or general ward without anesthesiologists.

Nara Medical University Hospital is a 992-bed tertiary care referral center with 15 operation rooms, including 1 hybrid operation room. Anesthesia information was obtained from our database, which had data provided by each anesthesiologist. We reviewed both electronic medical records and diagnosis procedure combination systems and extracted all patients who died by the first discharge after surgery. Among these patients, we identified those who died within 48 h of anesthetics.

### Definition of perianesthetic death

Perianesthetic death was defined as death within 48 h of an anesthetic [2]. Perianesthetic death was divided into the following three categories: anesthesia-related death (death solely attributable to either the anesthesia provider or the anesthetic technique), anesthesia-contributory death (death in which the anesthesia role could not be entirely excluded), and nonanesthesia-related death [3]. In cases of anesthesia-related death and anesthesia-contributory death, we identified the pathophysiological processes that best described the sequence of events [8]. The classifications of death and pathophysiological processes were performed independently by two researchers (MI and YN), and any disagreement was resolved by discussion with another researcher (MK).

### Explanatory variables

Data on patient characteristics, including sex, age, American Society of Anesthesiologists-Physical Status (ASA-PS), surgery circumstance (elective or emergency), surgery type, and anesthesia type, were collected. The surgery type was categorized according to the classification provided by the Japanese Society of Anesthesiologists. Burn surgery appears to be a high-risk procedure, but it is difficult to classify with the existing classification. Therefore, we added “burn surgery” to the existing classification. The anesthesia type was categorized as follows: general anesthesia with inhalation anesthesia, general anesthesia with total intravenous anesthesia, spinal anesthesia, epidural anesthesia, combined spinal and epidural anesthesia, and peripheral nerve block.

### Statistical analysis

The details of patients who experienced anesthesia-related death and anesthesia-contributory death are summarized. The incidence rate is expressed as occurrence in 10,000 anesthetics. The Wilson method was used to calculate 95% confidence interval (CI). In patients who experienced perianesthetic death, explanatory variables were analyzed using the Mann-Whitney *U* test or Fisher’s exact test. Patient age was categorized as follows: < 1 year, 1 to < 3 years, 3 to < 6 years, 6 to < 20 years, 20 to < 65 years, 65 to < 75 years, and ≥ 75 years. All analyses were performed using SPSS version 22.0 (IBM Corp., Armonk, NY, USA). The level of statistical significance was set at a *p* value < 0.05.

## Results

**Fig. 1:**
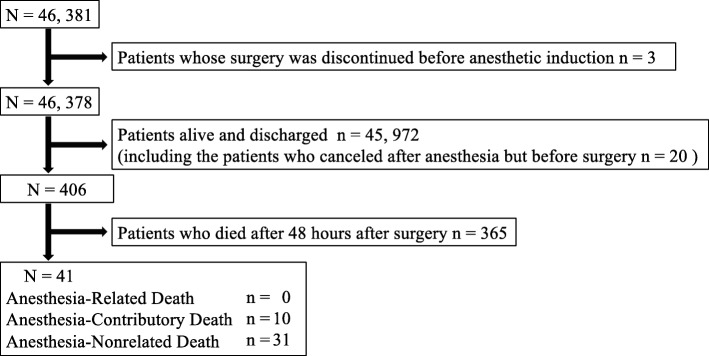
Patient flowchart

During the 10-year study period, 46,378 anesthetics were performed in our hospital. Of 406 patients who died by the first discharge after surgery, 41 experienced perianesthetic death, with an incidence of 8.8/10,000 anesthetics (95% CI, 6.1–11.6) (Fig. 1, Table 1). As shown in Table 1, the rate of perianesthetic death was high in the elderly, patients undergoing emergency surgery, and patients with a high ASA-PS. There was no anesthesia-related death. On the other hand, there were 10 anesthesia-contributory deaths, with an incidence of 2.1/10,000 anesthetics (95% CI, 0.69–3.6), and 31 nonanesthesia-related deaths, with an incidence of 6.8/10,000 anesthetics (95% CI, 4.2–9.1) (Table 2). Among the 41 patients, nonanesthesia-related death was more common in those undergoing emergency surgery (*p* = 0.04). Table 3 presents the details, including the pathophysiological mechanisms associated with the 10 anesthesia-contributory deaths. Overall, the events associated with anesthesia-contributory death were hypovolemia, myocardial infarction, arrhythmia, and respiratory failure. These events occurred during anesthesia maintenance in five 5 and after surgery in 5 patients.

**Table 1:** Patients’ background of the patients’ experienced perianesthetic death or not

	Perianesthetic death (−) (*n* = 46,337)	Perianesthetic death (+) (*n* = 41)
Age (years)	59.00 [36.00, 71.00]	73.00 [59.00, 81.00]
< 1	863 (1.9)	2 (4.9)
1 ≦, < 3	923 (2.0)	0 (0.0)
3 ≦, < 6	905 (2.0)	0 (0.0)
6 ≦, < 20	2822 (6.1)	2 (4.9)
20 ≦, < 65	22,403 (48.3)	9 (22.0)
65 ≦, < 75	10,777 (23.3)	11 (26.8)
75 ≦	7644 (16.5)	17 (41.5)
Male	21,597 (46.6)	29 (70.7)
ASA-PS		
I	14,514 (31.3)	0 (0.0)
II	26,880 (58.0)	0 (0.0)
III	4628 (10.0)	5 (12.2)
IV	298 (0.6)	12 (29.3)
V	17 (0.0)	24 (58.5)
Emergency surgery	7475 (16.1)	37 (90.2)
Type of surgery		
Neuro	4162 (9.0)	10 (24.4)
Thoracic	2247 (4.8)	2 (4.9)
Cardiac	1986 (4.3)	12 (29.3)
Esophageal	211 (0.5)	1 (2.4)
Upper abdominal	4216 (9.1)	1 (2.4)
Lower abdominal	9912 (21.4)	11 (26.8)
Cesarean section	3187 (6.9)	1 (2.4)
Head, neck, pharyngeal, and laryngeal	7137 (15.4)	0 (0.0)
Chest wall abdominal wall and perineum	2344 (5.1)	0 (0.0)
Spine	2113 (4.6)	0 (0.0)
Hip joint and limbs	6120 (13.2)	1 (2.4)
Examination	671 (1.4)	0 (0.0)
Electroconvulsive therapy	973 (2.1)	0 (0.0)
Others	724 (1.6)	0 (0.0)
Burn	316 (0.7)	0 (0.0)
Cancel after anesthesia induction	18 (0.0)	2 (4.9)
Type of anesthesia		
General anesthesia with inhalation anesthetics	31,894 (68.8)	41 (100)
General anesthesia with TIVA	9720 (21.0)	0 (0.0)
Spinal anesthesia	4122 (8.9)	0 (0.0)
Epidural anesthesia	420 (0.9)	0 (0.0)
Combined of spinal and epidural anesthesia	142 (0.3)	0 (0.0)
Peripheral nerve block	39 (0.1)	0 (0.0)

*ASA*-*PS* American Society of Anesthesiologists-Physical Status, *TIVA* total intravenous anesthesia

**Table 2:** Backgrounds of patients who experienced nonanesthesia-related death and those who experienced anesthesia-contributory death

	Nonanesthesia-related death (*n* = 31)	Anesthesia-contributory death (*n* = 10)	*p* value
Age (years)	73.00 [53.5, 81.00]	74.50 [61.75, 81.75]	0.54
< 1	2 (6.5)	0 (0.0)	0.84
1 ≦, < 3	0 (0.0)	0 (0.0)	
3 ≦, < 6	0 (0.0)	0 (0.0)	
6 ≦, < 20	1 (3.2)	1 (10.0)	
20 ≦, < 65	7 (22.6)	2 (20.0)	
65 ≦, < 75	9 (29.0)	2 (20.0)	
75 ≦	12 (38.7)	5 (50.0)	
Male	22 (71.0)	7 (70.0)	1
ASA-PS			0.4
I	0 (0.0)	0 (0.0)	
II	0 (0.0)	0 (0.0)	
III	2 (6.5)	3 (30.0)	
IV	10 (32.3)	2 (20.0)	
V	19 (61.3)	5 (50.0)	
Emergency surgery	30 (96.8)	7 (70.0)	0.04
Type of surgery			0.57
Neuro	10 (32.3)	0 (0.0)	
Thoracic	2 (6.5)	0 (0.0)	
Cardiac	7 (22.6)	5 (50.0)	
Esophageal	0 (0.0)	1 (10.0)	
Upper abdominal	0 (0.0)	1 (10.0)	
Lower abdominal	8 (25.8)	3 (30.0)	
Cesarean section	1 (3.2)	0 (0.0)	
Head, neck, pharyngeal, and laryngeal	0 (0.0)	0 (0.0)	
Chest wall abdominal wall and perineum	0 (0.0)	0 (0.0)	
Spine	0 (0.0)	0 (0.0)	
Hip joint and limbs	1 (3.4)	0 (0.0)	
Examination	0 (0.0)	0 (0.0)	
Electroconvulsive therapy	0 (0.0)	0 (0.0)	
Others	0 (0.0)	0 (0.0)	
Burn	0 (0.0)	0 (0.0)	
Cancel after anesthesia induction	2 (6.5)	0 (0.0)	
Type of anesthesia			1
General anesthesia with inhalation anesthetics	31 (100)	10 (100)	
General anesthesia with TIVA	0 (0.0)	0 (0.0)	
Spinal anesthesia	0 (0.0)	0 (0.0)	
Epidural anesthesia	0 (0.0)	0 (0.0)	
Combination of spinal and epidural anesthesia	0 (0.0)	0 (0.0)	
Peripheral nerve block	0 (0.0)	0 (0.0)	
General anesthesia	31 (100)	10 (100)	1
Pathophysiological process of death			0.26
Respiratory	1 (3.2)	1 (10.0)	
Arrhythmia	2 (6.5)	1 (10.0)	
Myocardial infarction	4 (12.9)	2 (20.0)	
Hypovolemia	13 (41.9)	6 (60.0)	
Central neurologic disorders	11 (35.5)	0 (0.0)	

*ASA-PS* American Society of Anesthesiologists-Physical Status, *TIVA* total intravenous anesthesia

**Table 3:** The pathophysiological mechanisms and details of patients who experienced anesthesia-contributory death

Number	Age (year)	ASA-PS	Emergency	Sex	Period of the event leading to death	Pathophysiological process	Detailed description	Location of the event leading to death
1	78	V	Yes	Male	Maintenance	Hypovolemia (hemorrhage)	Ruptured abdominal aortic aneurysm; intraoperative massive hemorrhage and bradycardia	Operating room
2	92	IV	No	Female	Postoperative period	Hypovolemia (true hypovolemia)	Bowel obstruction due to transverse colon cancer; atrial fibrillation with tachycardia and a large amount of intestinal solution	General ward
3	75	III	No	Male	Postoperative period	Hypovolemia (true hypovolemia)	Hemodialysis; postoperative hyperkalemia; hemodialysis difficulty due to hypovolemia	General ward
4	83	V	Yes	Female	Postoperative period	Hypovolemia (hemorrhage)	Ruptured abdominal aortic aneurysm; hypotension shortly after anesthesia induction; massive hemorrhage	Intensive care unit
5	48	V	Yes	Male	Postoperative period	Hypovolemia (sepsis)	Multiple organ failure and hypotension after replacement of the ascending aorta	Intensive care unit
6	70	V	Yes	Male	Maintenance	Hypovolemia (hemorrhage)	Ruptured abdominal aortic aneurysm; intraoperative massive hemorrhage	Operating room
7	59	III	No	Male	Maintenance	Myocardial infarction	History of angina pectoris; intraoperative ventricular fibrillation following ST-elevation; possibility of cardiac ischemia	Operating room
8	74	V	Yes	Female	Maintenance	Myocardial infarction	Aortic dissection with mitral valve regurgitation; low output syndrome; myocardial infarction on cardiology	Intensive care unit
9	12	III	Yes	Male	Maintenance	Arrhythmia	Perforation of a stress ulcer; burn; ventricular fibrillation caused by hyperkalemia after transfusion	Intensive care unit
10	88	IV	Yes	Male	Postoperative period	Respiratory failure	Bladder hemorrhage and anemia with multiple comorbidities; hypoxia after surgery	Operating room

*ASA-PS* American Society of Anesthesiologists-Physical Status

## Discussion

In the present study, the overall incidence of perianesthetic death was 8.8/10,000 anesthetics (95% CI, 6.1–11.6), whereas anesthesia-related death was not detected. Additionally, the incidence of anesthesia-contributory death was 2.1/10,000 anesthetics (95% CI, 0.69–3.6), and it was associated with hypovolemia, cardiac infarction, arrhythmia, and respiratory failure and was more common in elective surgery.

Previous studies have reported perianesthetic mortality using different definitions. Kawashima et al. used the definition of mortality occurring in the operating room and within seven postoperative days with accompanying critical events during surgery, whereas Lagasse et al. and Pollard et al. used the definition of death occurring within 48 h of anesthesia induction [2, 3, 7]. Our study used the latter definition, and the incidence of perianesthetic death (8.8/10,000) was similar to that reported in a recent study (7.5/10,000) [3]. Furthermore, as expected, age, emergency surgery, and high ASA-PS were associated with perianesthetic death, and these findings are consistent with the findings of previous studies [3, 8, 10, 11].

Fortunately, there was no anesthesia-related death during the 10-year study period. This might be because our sample size was limited and there were no perianesthetic deaths associated with failed ventilation, aspiration of gastric contents, and accidental bolus of narcotics, which can lead to anesthesia-related death and anesthesia-related cardiac arrest [3–6].

In the present study, 10 patients experienced anesthesia-contributory death, with an incidence of 2.1/10,000 anesthetics, and this incidence is higher than that reported in a study from the USA (0.22/10,000) [3]. This difference might be associated with differences in the study populations, including a smaller denominator, and the definitions of anesthesia-related death and anesthesia-contributory death. In addition, there was no anesthesia-related death in the present study, resulting in an increase in the incidence of anesthesia-contributory death. Furthermore, the rate of emergency surgery was higher among patients who experienced nonanesthesia-related death than among those who experienced anesthesia-contributory death. This might be because patient status, such as preoperative comorbidity, has greater effects on death when compared with anesthesia.

Among the 10 patients who experienced anesthesia-contributory death, 4 pathophysiological processes were identified. Hypovolemia is one of the most common complications during anesthesia, and almost all cases are managed well. Hypovolemia caused by rapid massive hemorrhage and large leakage into the tissues can lead to serious consequences, although this is rare. Perioperative acute myocardial infarction rarely occurs in patients undergoing noncardiac surgery, but it has been shown to be strongly associated with in-hospital mortality [12]. One patient was suspected of ST-elevation myocardial infarction (case 7 in Table 3), and this patient had a history of angina pectoris. Another patient presented with acute myocardial infarction postoperatively (case 8 in Table 3), and this patient underwent aortic surgery owing to aortic dissection. Acute myocardial infarction might be caused by aortic dissection; however, this case was included in the anesthesia-contributory death group because we were unable to exclude inadequate oxygen delivery following perioperative low output syndrome. Arrhythmia caused by hyperkalemia after transfusion might be preventable with frequent blood analysis. The etiology of postoperative respiratory failure is unknown. However, it might be caused by preoperative multiple comorbidities, including anemia, heart failure, and kidney injury.

The limitations of this study include its representation of perianesthetic death from a single institution. Our institution is a 992-bed tertiary hospital and includes a trauma center, but there are no organ transplantation surgeries, except for kidney transplantations. The rate of perianesthetic death can vary depending on the institution and study population. Additionally, the data were evaluated retrospectively, and thus, we experienced missing data and analyzed limited data. Moreover, death and pathophysiological processes were classified independently by two researchers, and they consulted with another researcher at the time of disagreement. The decisions might be different for other anesthesiologists.

## Conclusion

The incidence of perianesthetic death in this study performed at a Japanese tertiary hospital over a period of 10 years was 8.8/10,000 anesthetics. Additionally, no patient experienced anesthesia-related death, 10 experienced anesthesia-contributory death, and 31 experienced nonanesthesia-related death. The most common event leading to anesthesia-contributory death was hypovolemia, followed by myocardial infarction, arrhythmia, and respiratory failure. In the future, a further study using a large database with exact information is required to analyze the epidemiology of perianesthetic death and reduce its rate.

## Data Availability

They are available as a spreadsheet file upon reasonable request.

## Abbreviations

ASA-PS: American Society of Anesthesiologists-Physical Status
CI: Confidence interval
